# Supplementary material for: HIV-Related Knowledge and Practices among Asian and African Migrants Living in Australia: Results from a Cross-Sectional Survey and Qualitative Study
Source: Int J Environ Res Public Health. 2023 Feb 28;20(5):4347. doi: 10.3390/ijerph20054347 (PMC10002009; doi:10.3390/ijerph20054347)
Supplement: Supplementary file 1 [file ijerph-20-04347-s001.zip › HIV paper - Supplementary File S1.pdf]

Supplementary File S1: Interview topic guide

Overall interview aims and approach

Our overarching research question is:

> *In relation to migrants born in South-East Asia, North-East Asia and Sub-Saharan Africa, what* ***factors influence access to, and experiences of,*** *sexual health and blood-borne virus* ***services*** *in Australia?*

Our data collection approach is based on grounded theory. Underlying this approach is a commitment to "remaining open to the portrayals of the world encountered" (Timonen, Foley, & Conlon, 2018). This 'openness' requires researchers to be mindful of how their beliefs, assumptions and practices can influence the nature and quality of the study.

Data will be collected through in-depth interviews. In undertaking these interviews:

Do	Don't
• Use open questions<br>• Sensitively probe for detail, explanation and clarification at appropriate junctures:<br>o What happened next?<br>o Can you tell me more about that?<br>o What do you mean when you say ….?<br>o Why do you think that?<br>o Can you give an example of that?	• Introduce concepts or explanations that aren't expressed or implied by the participant<br>• Make unnecessary interjections

The following questions are some suggested ways of approaching the interview but they are only a guide. The guide is not prescriptive and the interviewer should always be led by the participant's responses.

Opening/rapport building questions

- Please tell me your age and a little bit about where you are from and how long you have been in Australia.
- What was is it like coming to a new country?

Pre-entry testing

- Did you have a health check before coming to Australia? If so, please tell me what this experience was like. How did the process make you feel?

Motivations for accessing health services

- Before you came to Australia, what kinds of things would you see a doctor for (if at all)? Why? (NOTE: Remind participants that you are not asking for their specific medical history – just ask them to speak in general terms).
- Without going into your personal health issues, can you explain what situations might lead you to see a doctor in Australia? (NOTE: If there is a difference between pre- and post-migration help-seeking practices explore reasons)
- Would you ever go to the doctor if you didn't feel sick? Why/why not? (NOTE: Explore practices both in country of origin and Australia and seek explanations for any differences)

Service choice and perceptions

- If you have seen a doctor in Australia recently, can you tell me about what led you to choose that doctor?
- How do you think that doctor would react if a patient asked to be tested for sexually transmissible infections and blood-borne viruses?

Pre- and post-migration knowledge

- What comes to your mind when I say STIs or sexually transmissible infections?
- What comes to your mind when I say BBVs or blood-borne viruses?
- Before coming to Australia, what did you know about HIV? Can you describe if there is anything new that you learnt about HIV since coming to Australia? (NOTE: Probe about knowledge of treatment availability and effectiveness especially).
- Before you came to Australia, what did you know about hepatitis B and hepatitis C? Can you describe if there is anything new that you learnt about hepatitis B or hepatitis C since coming to Australia? (NOTE: If necessary, can probe about symptoms, transmission, treatment etc).
- Before you came to Australia, what did you know about illnesses like chlamydia or gonorrhoea? Can you describe if there is anything new that you learnt about chlamydia or gonorrhoea since coming to Australia? (NOTES: If necessary, can probe about symptoms, transmission, treatment etc).
- (NOTE: If participants suggest that they knew less about a particular STI/BBV <u>before</u> coming to Australia, explore why they think that's the case).

Relative importance

- Thinking about chlamydia, hepatitis B, hepatitis C and HIV, which do you think affects more migrants from your country of origin? Why do you say this?
- Thinking about chlamydia, hepatitis B, hepatitis C and HIV, which do you think is most serious? Why do you say this?

Personal relevance

- How relevant do you think the topic of sexual health and blood-borne viruses is to <u>*you and your*</u> health? Why do you think this?
- How relevant do you think the topic of sexual health and blood-borne viruses is to *other people from your birth country who are living in Australia*? Why do you think this? Do you think it has more, less or the same relevance to *people from your birth country who <u>haven't</u> migrated*?

Possible service improvements

- Has a doctor ever offered you an STI or BBV test in Australia? If so, how did it make you feel at the time? If not, how would you feel if a doctor offered you an STI or BBV test?
- How comfortable would you feel learning more about STIs and BBVs with other migrants from your country of origin? Why?

Closing

- How did you feel doing this interview?
- Do you have any questions about what we discussed?
- Thank participant
