# Supplementary material for: HIV-Related Knowledge and Practices among Asian and African Migrants Living in Australia: Results from a Cross-Sectional Survey and Qualitative Study
Source: Int J Environ Res Public Health. 2023 Feb 28;20(5):4347. doi: 10.3390/ijerph20054347 (PMC10002009; doi:10.3390/ijerph20054347)
Supplement: Supplementary file 1 [file ijerph-20-04347-s001.zip › ijerph-2236932-supplementary.pdf]

Table S1. Variables mapped against relevant survey questions and response options

Question	Responses	Variable name	Variable type	Variable components	Notes/definitions
How old are you? (Tick one)	18-29 years<br>30-39 years<br>40-49 years<br>50-59 years<br>60 years and over<br>Prefer not to answer	Age	Categorical (ordinal)	18-29; 30-39; 40-49; 50-59; 60+	N/A
How do you identify? (Tick all that apply)	Woman<br>Man<br>Non-binary person<br>Transgender<br>Cisgender<br>Other [open text]<br>Prefer not to answer	Woman	Categorical	Yes – woman	Any of the following responses: woman + no other option selected; woman + cisgender; woman + transgender
				No – man	Any of the following responses: man + no other option selected; man + cisgender; man + transgender
				No - non-binary*	Any of the following responses: man + woman; transgender and no other option selected; non-binary person and no other option selected
What is the postcode in which you live?	Open text	State	Categorical	Western Australia	Any 6000-6999 postcode, any online survey with an embedded source code related to Western Australia, or any print survey known to be received from a Western Australian project officer
				Queensland	Any 4000-4999 postcode, any online survey with an embedded source code related to Queensland, or any print survey known to be received from a Queensland project officer
				Victoria	Any 3000-3999 postcode, any online survey with an embedded source code related to Victoria, or any print survey known to be received from a Victorian project officer
				South Australia	Any 5000-5999 postcode, any online survey with an embedded source code related to South Australia, or any print survey known to be received from a South Australian project officer
				Other	Any Australian postcode from a state other than the four above
		SEIFADecile	Categorical (ordinal)	1-10	Determined by mapping postcodes to Socioeconomic Indexes for Australia (SEIFA) 2016. A decile of 1 indicates lowest 10% of areas (most disadvantaged).
What is your residency status in Australia? (Tick one)	Permanent resident/citizen<br>Temporary – student visa<br>Temporary – work visa	PermanentCitizen	Categorical	Yes – permanent/citizen	Permanent resident/citizen selected

	Temporary – holiday/tourist visa<br>Temporary – partner visa<br>Other [open text]<br>I prefer not to answer			No – non-permanent	Any of the temporary categories selected (or other if open text unable to be assigned to a predefined category)
In which country were you born?	Open text for print survey<br>Choice of 67 countries for online survey	BirthRegion	Categorical	Northeast Asia	Any of the following responses: China (mainland); China (Hong Kong or Macau); Japan; Mongolia; North Korea; South Korea; Taiwan
				Southeast Asia	Any of the following responses: Brunei; Burma (Myanmar); Cambodia; Indonesia; Laos; Malaysia; Philippines; Singapore; Thailand; Timor-Leste; Vietnam
				Sub-Saharan Africa	Any of the following responses: Angola; Benin; Botswana; Burkina Faso; Burundi; Cameroon; Cape Verde; Central African Republic; Chad; Comoros; Democratic Republic of Congo; Republic of Congo; Cote d'Ivoire; Equatorial Guinea; Eritrea; Eswatini; Ethiopia; Gabon; The Gambia; Ghana; Guinea; Guinea-Bissau; Kenya; Lesotho; Liberia; Madagascar; Malawi; Mali; Mauritania; Mauritius; Mozambique; Namibia; Niger; Nigeria; Rwanda; Sao Tome and Principe; Senegal; Seychelles; Sierra Leone; Somalia; South Africa; South Sudan; Sudan; Tanzania; Togo; Uganda; Zambia; Zimbabwe
How many years have you been living in Australia in total?	Open text	YearsInAus	Continuous	N/A	N/A
To whom are you sexually attracted? (Tick all that apply)	Women<br>Men<br>Non-binary people<br>Others [open text]<br>I prefer not to answer	MSM	Categorical	Men attracted to men	Any respondent allocated to variable 'Gender - Man' (see above) who responds 'Men' to sexual attraction question (even if other responses also chosen)
				Men attracted to women only	Any respondent allocated to variable 'Gender - Man' (see above) who responds 'Women' to sexual attraction question (and no other response chosen)
What are the main languages you speak at home?	Open text	English	Categorical	English spoken at home	English listed as one of the languages spoken at home
				English not spoken at home	English not listed as one of the languages spoken at home
Have you heard of HIV and/or AIDS? (Tick one)	Yes<br>No	HeardHIV	Categorical	Heard<br>Not heard	Anyone who answered 'No' excluded from subsequent analysis of HIV knowledge questions (KnowHIVTest, KnowViralLoad, KnowART, KnowPrEP)
	Yes<br>No	KnowHIVTest	Categorical	Correct	'No' response

Is an HIV test done whenever someone has a blood test in Australia? (Tick one)	I don’t know			Not correct	‘Yes’ or ‘I don’t know’ response
Is it safe to have sex without a condom with someone who has VERY LOW amounts of HIV in their blood? (Tick one)	Yes<br>No<br>I don’t know	KnowViralLoad	Categorical	Correct	‘Yes’ response
				Not correct	‘No’ or ‘I don’t know’ response
Are there any medicines that people can take BEFORE sex to protect themselves against HIV?	Yes<br>No<br>I don’t know	KnowPrEP	Categorical	Correct	‘Yes’ response
				Not correct	‘No’ or ‘I don’t know’ response; NOTE: Those who chose ‘Yes’ but then provided an incorrect name for the medicine were recoded ‘Not Correct’
If you think there is a medicine that people can take before sex to protect themselves against HIV, what is the name of the medicine? (Tick one)	The name of the medicine is: (open text)<br>I don’t know the name of the medicine	Know_Prep_Name	Categorical	Yes	Responses include: PrEP, pre-exposure prophylaxis, tenofovir, Truvada or other recognized chemical or brand name
				No	‘I don’t know’ or incorrect name given
Can a person have an STI without any symptoms? (Tick one)	Yes<br>No<br>I don’t know	STI_Know_Sympt	Categorical	Correct	‘Yes’ response
				Not correct	‘No’ or ‘I don’t know’ response
In the past twelve (12) months, how many people have you had sexual intercourse with (vaginal or anal)? (Tick one)	0<br>1<br>2 to 5<br>6 to 10<br>11 or more<br>I prefer not to answer<br>I can’t remember	AmtSexPartners1	Categorical (ordinal)	0; 1; 2-5; 6-10; 11+; Can’t recall	Anyone who answered ‘0’, ‘I prefer not to answer’ or ‘I can’t remember’ excluded from analysis for variables CommittedPartner, CondomUsed and NoCondomReason
		AmtSexPartners2	Categorical	One partner only	‘1’ response
				More than one partner	‘2 to 5’, ‘6 to 10’ or ’11 or more’ responses<br>The purpose of this variable is only to include those who indicated they were sexually active
Which of the following best describes the MOST		CommittedPartner	Categorical	Committed	‘Someone you are in a committed relationship with (e.g. husband / wife, boyfriend / girlfriend)’ response

RECENT person you had sex with? (Tick one)	Someone you are in a committed relationship with (e.g. husband / wife, boyfriend / girlfriend)<br><br>A casual sex partner<br><br>A sex worker			Not committed	'A casual sex partner' or 'A sex worker' responses
Did you use a condom the MOST RECENT time you had sex?	Yes<br>No<br>I can't remember	CondomUsed	Categorical	Used	'Yes' response
				Not used	'No'<br>'I can't remember' only included in basic descriptive analysis.
Why did you NOT use a condom the MOST RECENT time you had sex (Tick as many as apply)	My partner and/or I didn't have one<br>My partner and/or I couldn't afford one<br>My partner did not want to use one<br>I did not want to use one<br>My partner and/or I did not know where to get one<br>My partner doesn't like the way they feel<br>I don't like the way they feel<br>My partner or I was trying to get pregnant<br>It is against my or my partner's culture or religion<br>My partner and I don't have any illnesses that can be passed on through sex<br>My partner and I trust each other<br>Another reason (please specify)	NoCondom_Reason	Categorical	See column 2	Each response option was coded 1 if selected and 0 is not selected
When did you have your most recent test for HIV, hepatitis B, hepatitis C or any sexually transmitted infections (we call these STI and/or BBV tests)?	Less than 12 months ago<br>1 to 2 years ago<br>More than 2 years ago<br>I have never been tested<br>I don't know	TestedRecently	Categorical	Within last two years	'Less than 12 months ago' or '1 to 2 years ago' response
				Not within last two years	'More than two years ago', 'I have never been tested'<br><br>'I don't know' only included in basic descriptive analysis.

Note: it doesn't matter if the test was in Australia or another country<br><br>(Tick one)					
What was your MOST RECENT STI and/or BBV test for?<br><br>(Tick as many as apply)	HIV<br>Chlamydia and / or gonorrhoea<br>Syphilis<br>Hepatitis B and / or hepatitis C<br>I don't know – it was a blood test<br>I don't know – it was a urine test<br>I don't know – it was a blood and urine test<br>Other [open text]	HIVTest	Categorical	HIV test	'HIV' response (either alone or in combination with another response)
				Other test	'Chlamydia and/or gonorrhoea' or 'Syphilis' or 'Hepatitis B and / or hepatitis C' or 'I don't know – it was a urine test' response without 'HIV' also being chosen<br><br>Two 'I do not know – it was a blood test' option only included in basic descriptive analysis
Why did you NOT have an STI and/or BBV test in the last two years? (Tick as many as apply)	I did not do anything to put me at risk<br>I was too embarrassed<br>I could not afford extra tests<br>I didn't know where to get one<br>I was scared about the result<br>I don't like needles/blood tests<br>I did not have any symptoms<br>I did not have the time to get tested<br>I did not think it was important<br>I couldn't get to a service/clinic<br>Another reason (open text)	Various	Categorical	See column 2	Multiple responses permitted
What was the reason for your MOST RECENT STI and/or BBV test? (Tick as many as apply)	I was applying for permanent residency<br>I had a new sexual partner<br>I shared injecting equipment with someone<br>Something happened that may have put me at risk<br>I was pregnant and had a check up<br>I was getting contraception/birth control<br>My doctor/nurse suggested it<br>My doctor/nurse just did it<br>I wanted to know if I had a sexually transmitted infection or a blood-borne virus<br>I had symptoms<br>It was part of my regular health check<br>I like to get regular STI/BBV tests	Various	Categorical	See column 2	Each response option was coded 1 if selected and 0 is not selected

	It was a requirement for my work/study<br>Another reason (open text)				
How would you feel if a doctor in Australia offered you STI and BBV tests during an appointment without you requesting any of these tests? (Tick any that apply)	Offended – why are they asking me<br>Worried – do they think I have an illness?<br>Surprised – I wasn't expecting that<br>Okay – STI and BBV testing is normal<br>Relieved – now I don't have to asl for the tests<br>Embarrassed – I'd rather not talk about these things<br>Other (open text)	Various	Categorical	See column 2	Each response option was coded 1 if selected and 0 is not selected
If a close friend in Australia told you that they were going to get tested for STIs and BBVs, how would you feel? (Tick any that apply)	Fine – it's none of my business<br>Shocked – I didn't think they would need to get tested<br>Proud – it's a responsible thing to do<br>Supportive – I am here if they need my help<br>Worried – I hope they are okay<br>Disappointed – they must have done something wrong<br>Other (open text)	Various	Categorical	See column 2	Each response option was coded 1 if selected and 0 is not selected
Since January 2018, how many times have you visited any country outside of Australia? (Tick one)	0<br>1 to 2 times<br>3 to 4 times<br>5 to 6 times<br>7 or more times<br>I can't remember the number of times	OverseasTravel	Categorical	Travel	'1 to 2 times', '30 to 4 times', '5 to 6 times', '7 or more times' or 'I can't remember the number of times'
				No Travel	'0'<br><br>Anyone who answered '0' excluded from analysis for variable TravelCondomUsed
On any of the overseas visits since January 2018, did you have sexual intercourse with at least one person who lives outside of Australia? (Tick one)	Yes<br>No<br>I prefer not to answer	TravelCondomUsed	Categorical	Condom used	'Yes' response
				Condom not used	'No response'
